# Radiochemotheraphy-induced oral mucositis: Ectoin solution as a new treatment

**DOI:** 10.4317/jced.59110

**Published:** 2022-05-01

**Authors:** Adriana Fondevilla, Ana Serradilla, Elena Moreno-Olmedo, Kirill Matskov, María-José Belmonte, Antonio Sáez, María-José Acevedo, Escarlata López

**Affiliations:** 1 MD, PhD. Department of Radiation Oncology. Vithas La Milagrosa Hospital, GenesisCare Madrid, Spain; 2 MD. Department of Radiation Oncology. Vithas La Milagrosa Hospital, GenesisCare Madrid, Spain; 3 NR. Department of Radiation Oncology. Vithas La Milagrosa Hospital, GenesisCare Madrid, Spain

## Abstract

**Background:**

The current treatment for head and neck cancer involves radiotherapy, systemic therapy and surgery in a multidisciplinary approach. Unfortunately, cancer therapies can lead to local and systemic complications or side effects such as mucositis, which is the most common dose-dependent complication in the oral cavity and gastrointestinal tract. Mucositis can cause a considerably reduced quality of life in cancer patients already suffering from physical and psychological exhaustion. Moreover, radiotherapy interruptions due to toxicity can impact negatively in local control and survival. The main purpose of this study was to analyze patient satisfaction of Ectoin solution use in radiotherapy or radiochemotherapy-induced oral mucositis.

**Material and Methods:**

This is an institutional prospective analysis including 15 patients, conducted by two Spanish centers, between October 2019 and May 2020. Patients were treated with Ectoin solution during Radiotherapy and one month after the end of the treatment, three times per day. A seven-ítem Likert scale was used. We present our descriptive statistic regarding doctors and patients´s satisfaction.

**Results:**

Our results suggest that Ectoin solution relieves mucositis and is well tolerated by patients.

**Conclusions:**

We observed a favorable repercussion in the oral mucositis management and suggest a potential benefit of treating it.

** Key words:**Radiotherapy, oral mucositis, head and neck cancer, ectoine, oral care.

## Introduction

The current treatment for head and neck cancer (HNC) involves radiotherapy (RT), systemic therapy and surgery in a multidisciplinary approach. Treatment goals include cure, organ and function preservation, as well as maintain quality of life (QoL) in terms of reducing adverse effects.

Advances in RT technology, as the introduction of intensity-modulated RT (IMRT) (1,2), have been shown to reduce side effects like xerostomia which is the main long-term side effect of the standard RT. Despite the implementation of these advances, mucositis is one of the most severe acute side effects of HNC therapies and continues to occur in all these patients (3) being directly dependent on the total radiation doses administered. Mucositis decreased QoL due to solid and liquid food dysphagia, dysarthria and odynophagia. In addition, it could even represent a gateway for opportunistic infections, complicating cancer treatment and extending hospitalization. Moreover, radiotherapy interruptions due to toxicity can impact negatively in local control and survival.

Clinically, mucositis, begins to develop as erythema followed by focal areas of oral mucosal desquamation. If mucositis is prolonged and severe, mucosal integrity is breached, ulceration begins, and the patient starts to have a burning sensation. Then, a fibrinous exudate, or pseudomembrane, containing bacteria covers the ulcer. Bacterial colonization occurs approximately two weeks after therapy. Management of mucositis often requires narcotic analgesics, intravenous fluids and gastrostomy feeding and despite all these supports, this side-effect may lead to unplanned RT interruptions, thereby compromising oncological outcomes (4,5). In the final stage of mucositis, epithelial cells controlled by signals secreted by the extracellular matrix and healing is completed within 4 weeks after the final dose of RT. Unfortunately, even after full replenishment of the epithelium, the structure of the reconstituted submucosa differs from its pre-RT state.

Although several topical antialgics and antioxidant agents have been tested in the prevention and supportive care of oral mucositis, there is a lack of evidence recommendations.

In this scenario, an ectoine-based mouthwash has been developed. Ectoin solution contains an amino acid expressed by halophile bacteria that lives in extreme conditions, with the ability to stabilize membrane structure and proteins. Ectoin forms water layers on top of cell membranes or lipids, stabilizing these structures and supporting their fluidity.

Recently it was reported that ectoine can induce structural changes in DNA *in vitro* (6-8) and even has the potential to protect DNA from ionizing radiation (IR).

Based on these properties Ectoin solution has the potential to protect human epithelia from allergens, heat or dryness, being able to reduce and prevent oral mucositis decreasing inflammation and promoting oral mucosa hydration (8-10).

We report data from a short study to explore Ectoin solution treatment can improve mucositis as side effects of RT or Radio-Chemotherapy (CRT) in HNC.

## Material and Methods

An institutional prospective analysis was conducted by two Spanish centers, GenesisCare Murcia and Granada, between October 2019 and May 2020.

GenesisCare Clinical Leader Forum approved this study in September 2019, under the direction of two senior doctors and following Helsinki Declaration. All patients have signed an approved informed consent. Ectoin Solution is already commercialized in Spain.

Due to the small number of the sample, we have only made a descriptive analysis of it using the SPSS V.20 statistical package.

The main purpose of this pilot study was to analyze the degree of patient’s satisfaction in RT or CRT-induced oral mucositis with Ectoin solution use. We use the scale of the Radiation Therapy Oncology Group/European Organization Research and Treatment of Cancer (RTOG/ EORTC), (11) which establishes the existence of 5 mucositis degrees based on the intensity of the affectation, from the absence of changes with respect to the basal situation (Degree 0) to the appearance of ulceration and/or necrosis (Degree 4). Acute toxicity was defined as toxicities less than 90 days from the end of radiation treatment.

Inclusion criteria were: 1) age older than 18 years; 2) HNC histologically proven; 3) Not demonstrated allergies.

Exclusion criteria were: 1) Allergy to Ectoin Solution; 2) Rejection of participate in the study.

A group of 15 patients who had HNC with larynx, oral cavity, oropharynx, nasopharynx cancer and cervical node metastases were included (Table 1). All patients underwent therapy with IMRT with simultaneous integrated boost (IMRT-SIB) under routine clinical practice. The primary tumor and involved lymph nodes were treated with 69.96 Gy in 33 daily fractions given Monday to Friday, 60Gy in 30 fractions was delivered to postoperative patients unless there was a macroscopic residual disease in which case 66Gy in 30 fractions was given. The medium risk volume received 59,4 Gy and low risk nodal levels 54,12Gy in 33 fractions or 60Gy and 54Gy in 30 fractions respectively with or without concurrent chemotherapy. If chemotherapy was indicated, Cisplatine 100mg/m2 every 21 days was the used regimen, in case of contraindication the alternative was Cetuximab, both administered concomitant with RT.

**Table 1:**
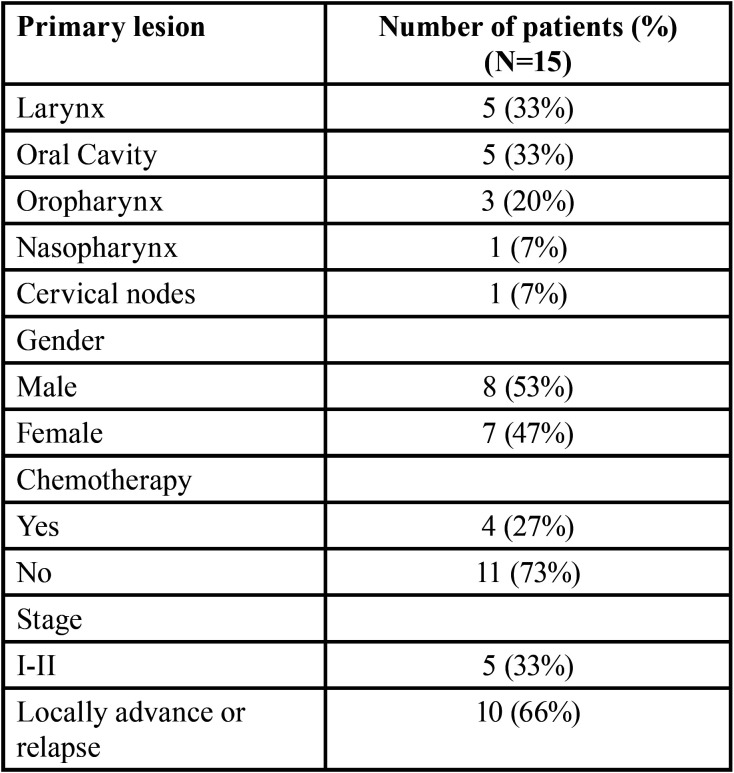
Baseline patients and tumor characteristics.

RT was administered using 6-MV photon beams through a linear accelerator in both Centers (ELEKTA SYNERGY® SLi; Elekta AB, Stockholm, Sweden).

According to the symptomatic control protocol of our clinics in the cases of HNC, a weekly evaluation was made by nurse, nutritionist and treatment physician.

All patients were treated with Ectoin solution for mucositis and xerostomia-related to radio and chemotherapy treatments. Ectoin solution was prescribed when the patient referred symptoms and the physical exploration revealed mucosal initial damage.

Ectoin solution solution is a registered medical device manufactured by Bad Homburg, Germany. It was given when grade ≥1 of xerostomia or mucositis toxicity appeared and patients expressed dryness, sore or pained oral cavity in despite of their medical treatment including opioids. Patients must rinse their mouth with an ampoule what contains 5 ml of solution, administered it 2 or 3 times every day depending on improvement, and for 1 hour after application they mustn’t eat or drink anything. The recommend use is to rinse the mouth for 2 minutes and throw the rest of liquid.

**Figure 1:**
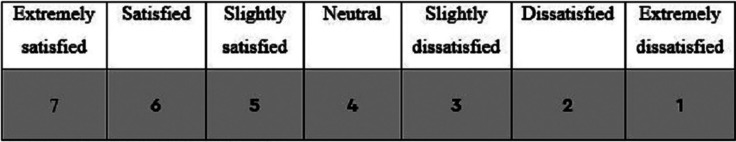
A seven-item L ikert sacle.

Follow up was two months after Ectoin solution treatment or clinical improvement, we evaluated the results and completed an internal satisfaction questionnaire from our institution which consist in two Likert satisfaction scales (12); one for patients and one for professionals, 1 and 2 months after the Ectoin solution treatment finished. The scale has seven items: 7: Extremely satisfied; 6: Satisfied; 5: Slightly satisfied; 4: Neutral; 3 Slightly dissatisfied; 2: Dissatisfied and 1: Extremely dissatisfied (Fig. 1).

This 7-item survey assessed patients’ global satisfaction in terms of perceived tolerability, acceptability of taste, ease or difficulty of application and overall experience when using the solution. From the professional point of view, the scale evaluated the patients ´adherence to the mouthwash and regarded acceptance or refusal for future prescription of ectoine solution.

## Results

Between October 2019 and May 2020, fifteen patients with HNC diagnosis were enrolled in the study. Patients´ clinical characteristics are summarized in Table 2.

**Table 2:**
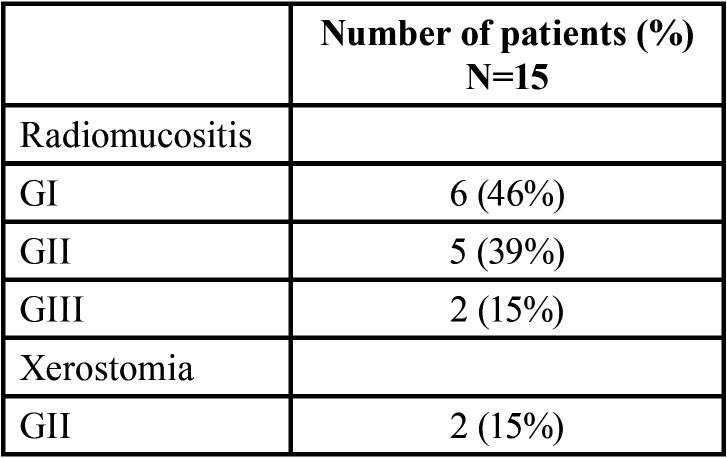
RT or CRT-induced acute toxicity.

Eight patients were male (53%) while 7 patients were female. Median age was 66 years (range, 37-82). The location of primary tumors was Oral-cavity (33%), Larynx (33%), pharyngeal (20%), Cavum (7%) and Cervical node (7%). The histology was 93% Squamous (SCC) and 7% Lymphoepithelioma. All patients had an Eastern Cooperative Oncology Group (ECOG) performance status 0-2 now of starting RT. Most of them had locally advanced stages or relapsed diagnosis (66%).

All patients were treated with IMRT-SIB with radical or adjuvant intention with or without systemic therapy (N=4; 27% vs N=11; respectively), including either cetuximab (N=1; 7%) or a platinum-based chemotherapy (N=3; 20%). Mean dose of RT was 68Gy (66-69.96Gy)

Mucositis grade 1 and 2 were observed in 46% (N=6) and 39% (N=5), respectively, while xerostomia grade 2 was seen in 15% (N=2). Most of patients, 13 (86%), needed opioid treatment for pain, but none required a feeding tube. No patient suspended radiotherapy treatment.

Table 3 shows toxicity and satisfaction questionnaire for patients as well as for professional experience.

**Table 3:**
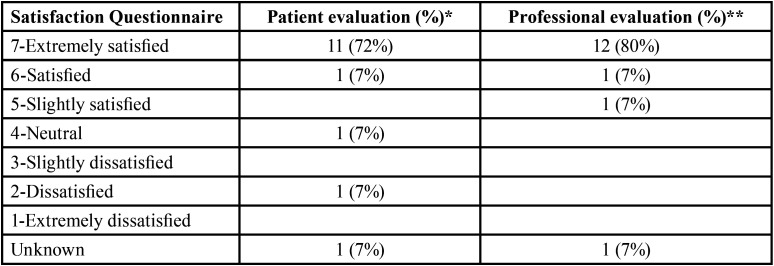
Satisfaction questionnaire for patients as well as for professional experience.

**Figure 2:**
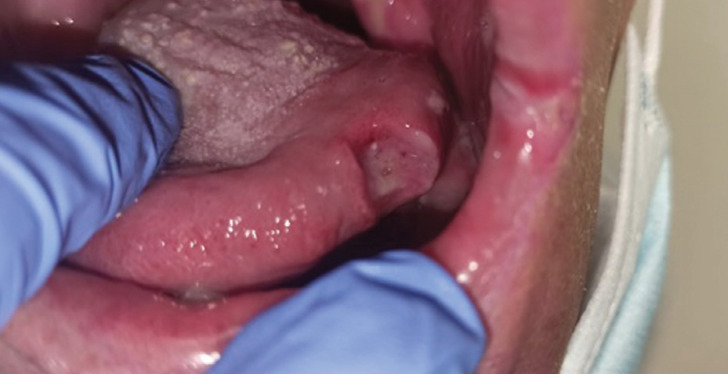
Patient with oral cavity cancer, two weeks evolution after Chemo-Radiotherapy with Mucavi® solution.

**Figure 3:**
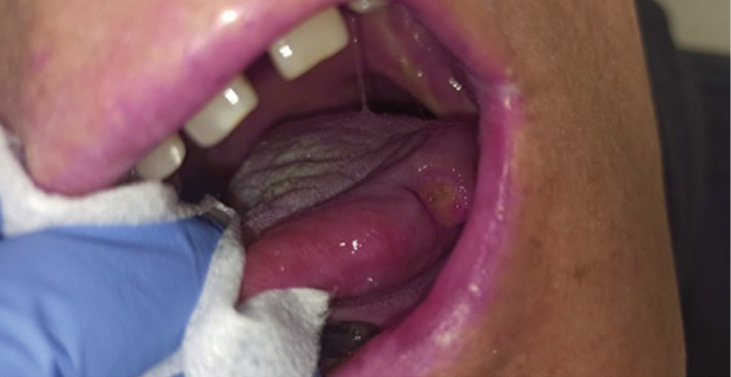
Patient with oral cavity cancer, one month evolution after Chemo-Radiotherapy with Mucavi® solution.

With a follow up of two months, eleven patients (73%) experienced a great improvement in mucositis expressed being “extremely satisfied” according to the Likert questionnaire. Regarding professional’s opinion, it was also an extremely good experience (80% of professionals) being parallel to the outcome of patients, (Figs. 2,3).

## Discussion

Our results show that Ectoin solution is an extremely satisfactory treatment (73% of the patients and 80% of the professionals) in terms of perceived tolerability, acceptability of taste, ease of application, overall experience, patients ´adherence and regarding acceptance of future prescription of ectoine solution.

Treatment for HNC entails high complexity, for this reason multidisciplinary tumor (MDT) board is essential to choose not only the best therapeutic approach but also the supportive measures (13).

Since IMRT emerged in 1980, it quickly gained popularity and became a standard RT technique for several types of tumors. IMRT is particularly favorable for irregular target volumes, located in a close proximity to organs at risk (OARs) (1,14), especially for HNC anatomy. One advantage of IMRT is to deliver high doses to the target volume with decreased OARs exposure, which means less side-effects.

Despite these technological advances, MDT decision and specialist team support, mucositis occurs, to a greater or lesser degree, in 100% of HNC patients.

Mucositis comprises progressive pain, increased mucous production, dry mouth, altered taste and edema. This may result in weight loss because of decreased oral intake of food, liquids and medication, like analgesics. Many patients need nutritional support and sometimes require a feeding tube with worse QoL in consequence (13). All these efforts focused on preventing treatment interruptions that would have a negative impact in local control and survival.

Mucositis treatment is challenging. Current supportive measures to reduce its risk and severity include: improvement of oral hygiene, which eliminates the presence of any irritants to the oral mucosa, systemic analgesics to control pain including narcotics (15) and several topical antalgic agents like GelClair® (Rubio Laboratory), GelX® (Ferrer laboratory) and Colutex® (Sativa laboratory).

A combination of treatments, such as local rinses with a 2% viscous lidocaine solution, a topical morphine solution and other systemic analgesics are also used to control pain (16). Rinsing with sodium chloride solution helps to keep the mucosa moist, reduces caking of secretions and soothes inflammation. However, there is no significant evidence to suggest that these mouthwashes are effective (3).

Moreover, several antioxidant agents to prevent mucositis or to reduce its severity have been tested. One of the first drugs used to treat mucositis was Amisfostine (17), but given its inconsistent results is no longer recommended for the oral mucositis prevention.

Glutamine is an amino acid used for oral mucositis during RT or chemotherapy but also came out with controversial results (18,19).

Low-level laser therapy (LLLT) is the technique that has shown the greatest effectiveness (20), but its evidence is moderate.

Due to the lack of evidence-based recommendations on mucositis support measures, we present the results of a prospective study to assess the relief of ectoine-based mouthwash in RT or CRT-induced oral mucositis by a Likert patient and professional satisfaction questionnaire.

Inspiring our study, recently it was reported that ectoine can induce structural changes in DNA *in vitro* (21) and even has the potential to protect DNA against IR.

Moreover, a prospective study has already showed effectiveness and tolerance with Ectoin versus calcium phosphate mouthwash for the treatment of mucositis following Chemotherapy. Indeed, patients preferred Ectoin to calcium phosphate mouthwash (8).

For our knowledge this is the first pilot clinical study which evaluates ectoine effects in RT or CRT-induced mucositis.

According to some clinical analyses 30-50% of HNC patients needed nutritional supports (22) and 60% required a feeding tube (23) during the oncological treatments. In our series, although most patients needed opioids (80%), no one required a feeding tube or was hospitalized. Because of that, we support nursing, treating physicians and nutritionist consultation weekly during the treatment are key in the management of care and RT adherence for patients with mucositis-xerostomia CRT-related.

Furthermore, avoiding or reducing the severity of mucositis has a positive impact on the adherence of treatment, preventing delays in treatment that may adversely affect the oncological HNC outcomes. In our data, no patient suspended radiotherapy treatment.

Conclusions from this review should be guarded given several limitations of this study. The number of patients included in this study is small (15 patients), the cohort is heterogeneous for histology and tumor location, and also, there is no control arm. If we analyze by subgroups, they are unbalanced: only 4 (27%) patients were treated with concomitant CRT and 73% with exclusive RT. Furthermore, five patients (33%) had early HNC and 66% had relapsed or locally advanced HNC. We must consider that the incidence of oral mucositis varies depending on the location of the primary tumor, being higher in the oral cavity, oropharynx, and nasopharynx, in the patients who receive concurrent CRT and in who received a higher total dose.

We emphasize the importance of performing clinical usage of CTCAE or RTOG criteria, to assess the response to treatment as accurately as possible.

Because we have prescribed ectoin when the patients were symptomatic, we wonder if the prophylactic use of the ectoin mouthwash may be beneficial, so we encourage further studies to evaluate the preventive role of ecotine solution, as well as studies with a longer follow up.

Despite these limitations, we observed a favorable repercussion in the oral mucositis management and suggest a potential benefit of treating oral RT or CRT-induced mucositis with Ectoin solution because of the impact in patient and professional´s satisfaction (both >70% extremely satisfied) and no one suspended RT treatment.

## Conclusions

Ectoin solution is an active agent for RT and CRT-induced oral mucositis, easy to apply and well tolerated. We observed a favorable repercussion in the oral mucositis management and suggest a potential benefit of treating it.

Further studies and longer follow-up could demonstrate the effectiveness of Ectoin solution, in terms of evaluating the impact in long-term side effects of CRT induced mucositis.

Mucositis grade 1 and 2 were observed in 46% (N=6) and 39% (N=5), respectively, while xerostomia grade 2 was seen in 15% (N=2). Most of patients, 13 (86%), needed opioid treatment for pain, but none required a feeding tube. No patient suspended radiotherapy treatment.

Table 2 shows RT or CRT-acute toxicity; Table 3 shows satisfaction questionnaire for patients as well as for professional experience.

With a follow up of two months, eleven patients (73%) experienced a great improvement in mucositis expressed being “extremely satisfied” according to the Likert questionnaire. Regarding professional’s opinion, it was also an extremely good experience (80% of professionals) being parallel to the outcome of patients.
